# A Hybrid Photoreceptor Expressing Both Rod and Cone Genes in a Mouse Model of Enhanced S-Cone Syndrome

**DOI:** 10.1371/journal.pgen.0010011

**Published:** 2005-08-05

**Authors:** Joseph C Corbo, Constance L Cepko

**Affiliations:** 1 Department of Genetics, Harvard Medical School, Boston, Massachusetts, United States of America; 2 Department of Pathology, Brigham and Women's Hospital, Boston, Massachusetts, United States of America; 3 Howard Hughes Medical Institute, Harvard Medical School, Boston, Massachusetts, United States of America; Stanford University School of Medicine, United States of America

## Abstract

Rod and cone photoreceptors subserve vision under dim and bright light conditions, respectively. The differences in their function are thought to stem from their different gene expression patterns, morphologies, and synaptic connectivities. In this study, we have examined the photoreceptor cells of the *retinal degeneration 7* *(rd7)* mutant mouse, a model for the human enhanced S-cone syndrome (ESCS). This mutant carries a spontaneous deletion in the mouse ortholog of NR2E3, an orphan nuclear receptor transcription factor mutated in ESCS. Employing microarray and in situ hybridization analysis we have found that the *rd7* retina contains a modestly increased number of S-opsin–expressing cells that ultrastructurally appear to be normal cones. Strikingly, the majority of the photoreceptors in the *rd7* retina represent a morphologically hybrid cell type that expresses both rod- and cone-specific genes. In addition, in situ hybridization screening of genes shown to be up-regulated in the *rd7* mutant retina by microarray identified ten new cone-specific or cone-enriched genes with a wide range of biochemical functions, including two genes specifically involved in glucose/glycogen metabolism. We suggest that the abnormal electroretinograms, slow retinal degeneration, and retinal dysmorphology seen in humans with ESCS may, in part, be attributable to the aberrant function of a hybrid photoreceptor cell type similar to that identified in this study. The functional diversity of the novel cone-specific genes identified here indicates molecular differences between rods and cones extending far beyond those previously discovered.

## Synopsis

Vision begins with light entering the eye. This light is projected onto the retina, a thin neural structure lining the inside of the eye. Photoreceptors, among the most important cell types in the retina, are the first to receive the incoming rays of light. In mammals, there are two types of photoreceptors: rods and cones. Rods are specialized for nighttime vision, and cones for daytime and color vision. In this study, the authors examined the photoreceptors of a mouse with a gene mutation that causes photoreceptors to develop abnormally. Humans with a similar mutation have a form of blindness called enhanced S-cone syndrome (ESCS). Surprisingly, the majority of photoreceptors in this mutant mouse were found to have features of both normal rods and cones. It is possible that the abnormal features of these photoreceptors predispose them to undergo premature death. If this model accurately reflects the situation in human patients with ESCS, it may provide an explanation for the loss of vision seen in this disease. This study also elucidated previously unknown molecular differences between normal rods and cones. This new knowledge may contribute to a better overall understanding of the mechanisms underlying night, day, and color vision.

## Introduction

Enhanced S-cone syndrome (ESCS) is an unusual disease of photoreceptors that includes night blindness (suggestive of rod dysfunction), an abnormal electroretinogram (ERG) with a waveform that is nearly identical under both light and dark adaptation, and an increased sensitivity of the ERG to short-wavelength light [1,2]. The disease is caused by mutations in the orphan nuclear receptor transcription factor NR2E3 (also known as photoreceptor nuclear receptor), which is expressed exclusively in rods [3,4]. Recent human genetic studies have also demonstrated mutations in this gene in Goldmann-Favre syndrome and many cases of clumped pigmentary retinal degeneration [5].

The initial reports of patients with ESCS attributed the unusual ERG to an abnormally functioning rod photoreceptor system with persistent activity under light adaptation [6–8]. Subsequent studies, however, concluded that the ERG was due to supernumerary short-wavelength (“blue”) cone photoreceptors (S-cones) in these patients [1,2,9–11]. Histopathologic analysis of a retina from a human patient with ESCS and extensive retinal degeneration demonstrated an absence of rhodopsin-positive cells and an increase in the number of S-cone opsin-expressing cells. Nevertheless, the overall density of cones was only modestly increased in this patient (approximately 2-fold), suggesting that there might be additional factors that contribute to the very large, light-adapted ERG seen in this disease. In addition to the ERG findings, patients with ESCS have dysmorphic retinas with rosette formation in the outer nuclear layer (ONL) where photoreceptor cell bodies reside, and a slow retinal degeneration that can ultimately lead to complete blindness [12–14].

Mutations in the mouse ortholog of NR2E3 have been identified in the spontaneous mutant *retinal degeneration 7* *(rd7)* [15]. This mutant demonstrates slow retinal degeneration and abnormal lamination of the ONL with rosette formation [15,16]. Curiously, the ERG of the mouse under both light and dark adaptation has been reported to be normal, showing progressive attenuation with time, presumably due to degenerative cell loss [15]. A prior study showed a 2- to 3-fold increase in the number S-opsin–positive cells in the *rd7* retina compared to wild type [17]. In addition, two groups recently reported derepression of additional cone genes in the *rd7* mutant [18,19].

In order to better understand the mechanistic basis of ESCS, we undertook a molecular and ultrastructural analysis of the photoreceptors of the *rd7* mutant mouse. Microarray and in situ hybridization analyses revealed a modest increase in the number of S-opsin–positive cells and widespread derepression of many cone-specific genes within rod photoreceptor cells. Ultrastructural studies demonstrated that the cells that coexpress rod and cone genes in the *rd7* retina represent a morphologically hybrid cell type, intermediate between normal rods and cones.

## Results

### Widespread Up-Regulation of Cone Genes in the *rd7* Mutant Retina

In an initial analysis of the *rd7* mutant, homozygous mutant retinas were compared with wild-type controls at multiple postnatal time points using both cDNA and Affymetrix microarrays. The cDNA microarray used in this study contains approximately 12,000 different cDNAs largely derived from the retina and nervous system, and the Affymetrix microarray contains over 34,000 genes. Experiments at all timepoints were carried out in triplicate, and stringent criteria were applied in deciding whether a given gene was up- or down-regulated in the mutant (see Materials and Methods for details).

**Figure 1:**
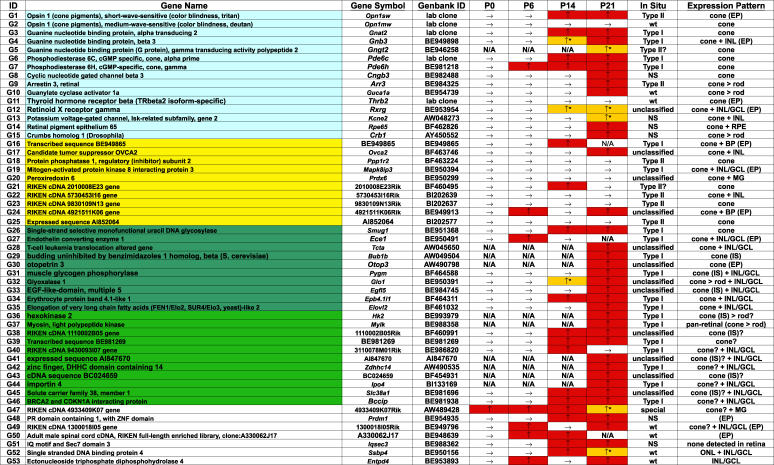
Cone-Specific and Cone-Enriched Genes Evaluated in the *rd7* Mutant by Microarray and In Situ Hybridization The color coding of text in the column “Gene Name” is as follows: light blue (G1–G15), genes previously reported in the literature to have cone-specific or cone-enriched patterns of expression; yellow (G16–G25), novel cone genes identified in an unrelated study (unpublished data); dark green (G26–G36), novel cone genes identified in the present study that were up-regulated in *rd7;* light green (G37–G46), additional genes found to be up-regulated in *rd7* by microarray in the present study but that had either weak or inapparent cone-specific signal on in situ hybridization; white (G47–G53), additional genes up-regulated by microarray at two different timepoints but with either unusual expression patterns or nonconfirmatory in situ hybridizations. The column “ID” contains identifiers used in the present paper to refer to specific genes. “GenBank ID” contains the GenBank accession number of the clone used to make the probe for in situ hybridization. Within this column, “lab clone” indicates that the probe used for in situ hybridization derived from a clone in our laboratory. The region of the gene to which it corresponds is indicated in Table S1. Columns “P0” through “P21” contain the results of microarray experiments at the given postnatal dates. P0, P6, and P14 time points represent analyses on cDNA microarrays; the P21 time point represents data from an Affymetrix microarray (mouse genome 432 2.0). A red cell with a single up arrow indicates that the gene in question was up-regulated in three out of three microarrays at that time point (as described in Materials and Methods). Those cells labeled orange with a single up arrow and asterisk indicate that the gene in question was up-regulated in two out of three microarrays at that time point. The column “In Situ” lists the type of derepression seen for the gene in question in the *rd7* mutant retina (type I and type II are described in the main text). Genes designated “unclassified” represent patterns of derepression that were difficult to classify as either type I or type II (see main text for more details). “Wild type” in this column indicates that the in situ hybridization pattern in the *rd7* mutant retina was not different from the wild-type pattern; and “special” indicates a special pattern of expression discussed more fully in the main text. The column “Expression Pattern” contains a concise description of the wild-type expression pattern of the gene in question. In the case of genes for which no signal was obtained on in situ hybridization in the present study, the specified expression pattern derives from reports in the literature. Within this column, “cone > rod” indicates that the gene is expressed in all photoreceptors, but at higher levels in cones than rods; “cone?” indicates very weak staining in a cone-like distribution. BP, bipolar cells; EP, early photoreceptor expression pattern; IS, inner core segment localization; MG, Müller glia; N/A, not available on the microarray; NS, no signal detected on in situ hybridization; RPE, retinal pigment epithelium.

These experiments demonstrated widespread up-regulation of cone-specific and cone-enriched genes in the *rd7* retina, especially by postnatal day 14 (P14) and P21 (Figure 1). Most known cone-specific or cone-enriched genes were found to be up-regulated in the mutant (Figure 1, genes G1–G15). The majority of these genes represent components of the phototransduction cascade (e.g., opsins, transducins, and phosphodiesterase subunits). In addition to these genes, several novel cone-specific genes of unknown function recently identified in our lab were also up-regulated (Figure 1, genes G16, G17, G21, and G24; unpublished data). Finally, a wide range of other genes, most with no previously recognized role in the retina, were found to be up-regulated in the *rd7* mutant (Figure 1, G26–G53; Tables S1 and S2; Figures S1–S7).

*Nr2e3* expression is first detectable by in situ hybridization around embryonic day 18 (E18); it then peaks around P6 and subsequently decreases to adult levels by P21 (unpublished data). In accordance with this time course of expression, almost no gene expression changes were found at P0, with progressively more changes at later timepoints (Figure 1). One exception to this statement is the gene RIKEN cDNA 4933409K07 (Figure 1, gene G47), which was the only gene shown to be up-regulated at all timepoints examined. Additional discussion of this gene and its unusual expression pattern will be presented below.

### Two Distinct Patterns of Cone Gene Derepression in *rd7*

**Figure 2:**
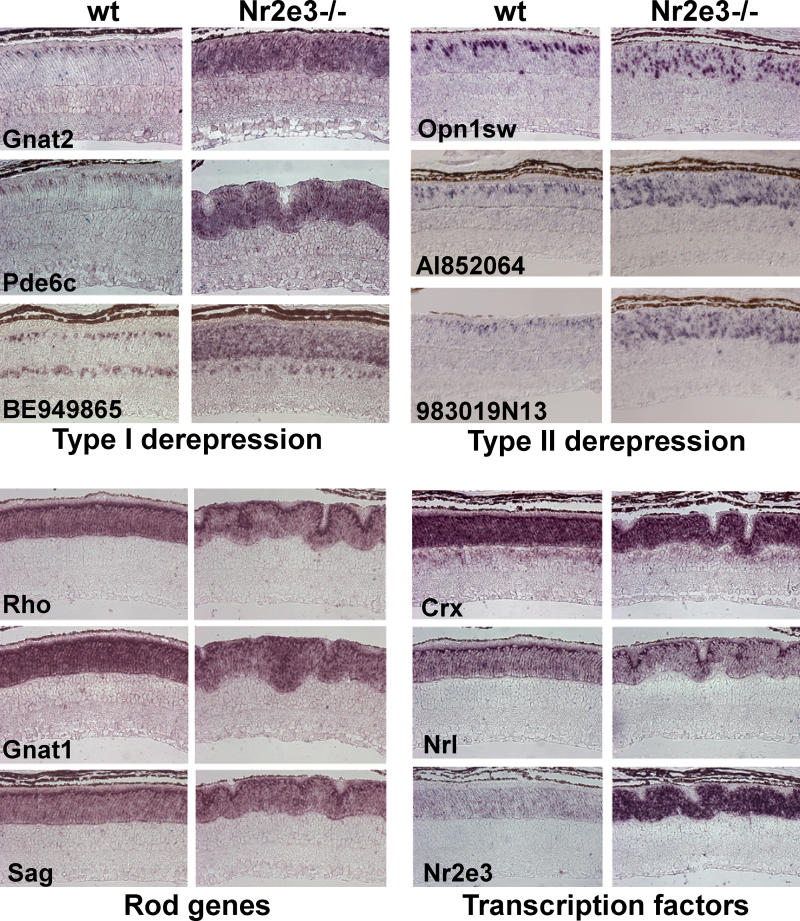
Cone and Rod Gene Expression in the *rd7* Mutant at P14 The upper sets of photomicrographs demonstrate examples of type I and type II cone gene derepression in the *rd7* mutant retina as explained in the main text. The bottom left images show several rod-specific genes that are essentially unchanged in the *rd7* background at P14. The bottom right images show the expression pattern of three photoreceptor transcription factors in the *rd7* mutant. Abbreviations in the lower left hand corner of each pair of panels represent the gene symbols summarized in Figure 1.

In order to confirm these microarray results, an in situ hybridization analysis of the putative up-regulated cone genes was carried out in which the *rd7* mutant retina was compared with age-matched, wild-type controls. We found that the majority of the cone-specific genes that were up-regulated in microarray experiments were derepressed when assessed by in situ hybridization (Figure 2). There were two major patterns of cone gene derepression. The more common pattern (type I) manifested itself as ectopic gene expression throughout the ONL, consistent with gene expression in all photoreceptors (Figure 2; upper left photomicrographs). Typical examples of this pattern of derepression are shown in Figure 2, and many more are available in Table S1. This pattern of expression contrasts sharply with the usual pattern of cone gene expression, which consists of scattered cells localized to the scleral edge of the ONL (Figure 2).

The second category of cone gene derepression (type II) consisted of a patchy, salt-and-pepper pattern of ectopic expression in which individual positive cells were scattered throughout the ONL (Figure 2, upper right photomicrographs; Table S1). Although numerous positive cells were present in the *rd7* retina (particularly in the ventral portion), there were clearly many interspersed cells that showed a complete absence of expression. In order to rule out the possibility that these scattered positive cells were simply the normal complement of cones that had failed to localize their cell bodies to the scleral edge of the ONL, the number of positive cells in the *rd7* retina was quantitated by dissociated cell in situ hybridization.

**Figure 3:**
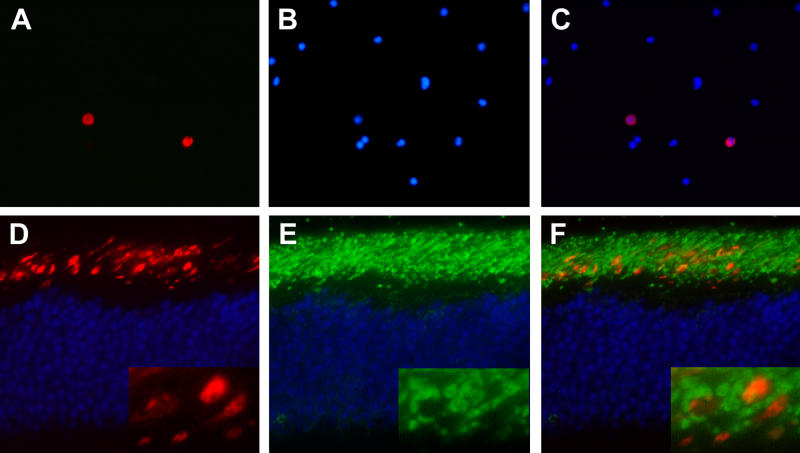
S-Opsin Dissociated Cell In Situ Hybridization and S-Opsin/Rhodopsin Antibody Staining on *rd7* Mutant Retina (A–C) A dissociated cell in situ hybridization with an S-opsin probe (red) on dissociated *rd7* mutant retinal cells stained with DAPI (blue). (C) shows the merged images. (D–F) The outer nuclear layer of an *rd7* mutant retina stained by antibody for S-opsin (red) and rhodopsin (green). The scleral edge of the outer nuclear layer is up. DAPI staining is in blue. (F) shows the merged images. Insets are higher-power images of the outer segments showing non-overlap of S-opsin and rhodopsin staining in the mutant.

Dissociated cell in situ hybridization was performed using a probe for the S-cone opsin gene *(Opn1sw),* which shows type II derepression (Figures 2 and 3A–3C). S-opsin was expressed in 3.2% of retinal cells in the *rd7* mutant (66 S-opsin–positive cells out of 2,056 6-diamidino-2-phenylindole [DAPI]-positive cells). This value is approximately 2-fold greater than the percentage of S-opsin–positive cells identified in wild-type control retinas, 1.65% (54 S-opsin-positive cells out of 3,271 DAPI-positive cells), and accords well with the previously reported value of 2- to 3-fold more S-opsin–positive cells in *rd7* compared to wild type arrived at by antibody staining of tissue sections [17].

Previous studies have estimated that the total number of cones in the mouse retina is 2% of all retinal cells [20], and that S-opsin is largely repressed in cones in the dorsal third of the retina [21]. The estimate of 1.65% S-opsin–positive cells in the wild-type retina is in agreement with these data. The fact that only 3.2% of all retinal cells are S-opsin–positive in the *rd7* mutant also confirms that the majority of the photoreceptors (which make up just over 70% of the cells in the adult mouse retina) do not express this gene. In order to assess whether these supernumerary S-opsin–expressing cells coexpressed rod-specific markers, a double antibody staining for S-opsin and rhodopsin was performed. This study showed mutually exclusive domains of expression of S-opsin and rhodopsin in the photoreceptor outer segments (Figure 3D–3F). This finding suggests that the supernumerary S-opsin–expressing cells in the *rd7* retina may represent normal “blue” cones.

### Novel Cone-Specific Genes Are Derepressed in *rd7*

**Figure 4:**
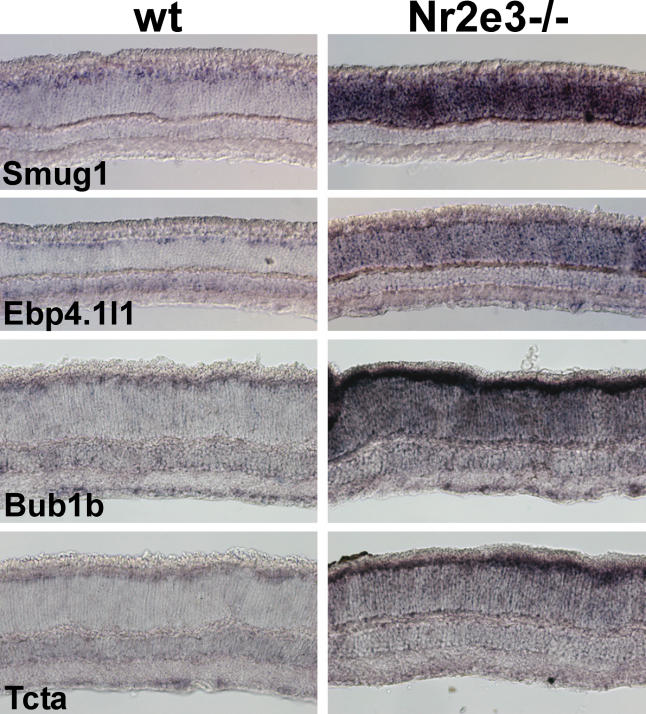
Expression Patterns of Several Novel Cone Genes Up-Regulated in *rd7* In the wild-type images (wt), note the scattered, weakly positive cells at the scleral edge of the outer nuclear layer in a cone distribution. All of the genes show marked up-regulation in the *rd7* mutant. *Bub1b* and *Tcta* show transcript localization predominantly to the inner segment of the photoreceptors. Retinas are oriented such that the scleral edge is up.

Given that the majority of known cone-specific genes showed marked derepression in the *rd7* mutant, additional candidate genes up-regulated on microarray analysis were evaluated for cone-specific expression. In situ hybridization was performed on an additional 45 up-regulated genes, confirming that 21 of them were derepressed. Of these, at least ten showed a definite cone-specific or cone-enriched pattern of expression in the wild-type retina (Figure 1, genes G26–G35). Several examples are given in Figure 4. Note that in the wild-type retina, there is a relatively weak pattern of scattered positive cells at the scleral edge of the ONL, consistent with a cone-specific pattern of expression. All of these genes show marked derepression in the *rd7* retina. A number of these novel cone-specific genes showed a striking localization of their transcripts to the photoreceptor inner segment (e.g., Bub1b and Tcta). This localization manifests in a section in situ hybridization as a dark band of staining just beyond the outer edge of the ONL immediately underlying the outer segment layer. Although such a pattern of transcript localization is commonly seen in many rod-specific genes (e.g., *Rho* in Figure 2; *Pcdh21,* *Rbp3,* and *Cnga1* in Table S2), it is not easily appreciated in cone-specific genes, possibly due to the relative scarcity of cones in the mouse. In the *rd7* mutant retina in which such genes are widely derepressed, such a pattern of transcript localization often becomes apparent.

In addition to the ten genes that showed cone-specific expression in the wild-type retina, another 11 novel genes were derepressed in the *rd7* retina by in situ hybridization (Figure 1, genes G36–G46). Some of these genes showed faint expression in a cone-like distribution (see Table S1, genes G36, G40, and G44), and one appeared to be expressed throughout the ONL but at greater levels in cones than in rods (Table S1, gene G37). The remainder of the up-regulated genes did not have detectable cone staining in the wild-type retina. Despite this apparent absence of cone staining, the pattern of derepression in *rd7* suggests that these genes may also be novel cone-specific genes, albeit expressed at levels below the sensitivity threshold of our in situ hybridization assay.

In most cases, the novel cone genes identified in this study appear to have a type I pattern of derepression. However, due to the weakness of the signal in some cases, or transcript localization to the inner segment in others, it was not always possible to determine with confidence which of the two patterns of derepression (if either) each of these genes displayed. In terms of functional categorization, the novel cone genes cover a broad range including glucose metabolism *(Pygm* and *Glo1),* fatty acid metabolism *(Elovl2),* DNA repair *(Smug1),* cell cycle/chromosome segregation *(Bub1b),* carcinogenesis *(Tcta),* endothelial biology *(Ece1),* cytoskeletal function *(Ebp4.1l1),* and even otolith formation *(Otop3)*.

**Figure 5:**
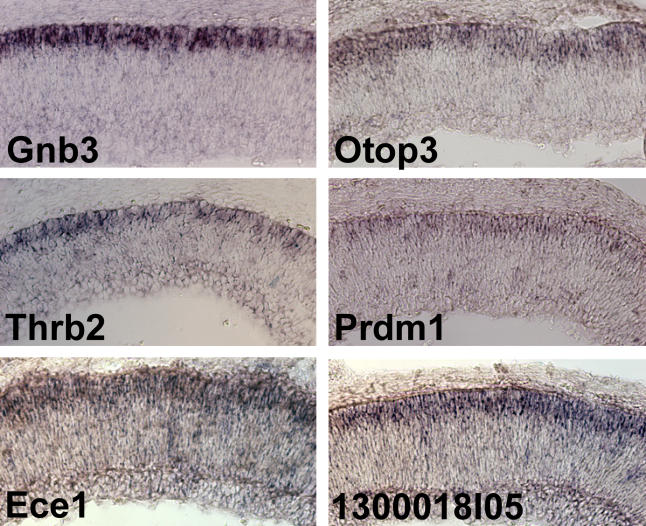
Some of the Genes Up-Regulated in *rd7* Show an Early Photoreceptor Pattern of Expression *Gnb3* and *Thrb2* are both previously characterized cone genes that show staining at the scleral edge of the embryonic mouse retina in cells that will differentiate into photoreceptors. *Ece1* and *Otop3* are two novel cone genes identified in this study that were up-regulated in *rd7* and also showed an early photoreceptor pattern of expression. *Prdm1* and RIKEN cDNA 1300018I05 (Figure 1, genes G48 and G49, respectively) are two other genes that had either undetectable signal *(Prdm1)* or no apparent change in expression pattern in adult *rd7* mutants (RIKEN cDNA 1300018I05), but which also showed staining in the embryonic retina in a presumptive photoreceptor pattern. All images are from E17.5 retina except *Gnb3,* which was from E16.

A relatively frequent finding among both previously identified cone-specific genes, as well as in some of those identified in the present study, is the occurrence of gene expression in an early photoreceptor precursor pattern (Figure 5). This pattern of expression consists of positive staining by in situ hybridization specifically at the scleral border of the retina during prenatal timepoints (in the range of E13–E18). *Gnb3* and *Thrb2* are two examples of known cone genes with this early pattern of expression (Figure 5). Two of the 11 novel cone genes identified in this study also have this early photoreceptor pattern of expression *(Ece1* and *Otop3)*. Intriguingly, three genes shown to be up-regulated in *rd7* on microarray, but that had either no detectable signal by in situ hybridization at adult stages or no apparent change in expression by in situ hybridization between wild type and *rd7,* also showed this early photoreceptor pattern (Figure 1, genes G48–G50). The embryonic expression pattern of two of these genes is shown in Figure 5 (the embryonic in situ hybridization for the third, G50, can be found in Table S1). Although the significance of such early photoreceptor expression is not known, it is possible that these genes may also be cone-specific but are expressed at undetectably low levels in the adult.

### M-Opsin and Thyroid Hormone Receptor β2 Are Unchanged in the *rd7* Mutant

Only two cone-specific genes failed to show any change in expression by either microarray or in situ hybridization in the *rd7* mutant: M-opsin *(Opn1mw)* and thyroid hormone receptor β2 *(Thrb2)* (Figure S8). This result is particularly notable because *Thrb2* is absolutely required for the expression of M-opsin [22]. Furthermore, the repression of S-opsin expression in the dorsal third of the mouse retina is thought to depend, at least in part, on Thrb2 since S-opsin shows dorsal derepression in the *Thrb2* mutant [22]. Despite the derepression of S-opsin seen in the ventral portion of the *rd7* retina, the normal dorsal repression of this gene is still present in this mutant (unpublished data). This finding is consistent with the normal expression pattern and function of *Thrb2* in the *rd7* mutant.

One further finding to note is that the cell bodies of the M-opsin–positive cells appear to be scattered throughout the ONL in the *rd7* mutant at P14 (Figure S8). Despite this fact, their overall number does not appear to be increased relative to wild-type. In addition, by P28, the M-opsin-positive cell bodies in *rd7* appear to have relocated to their normal position at the scleral edge of the ONL (Figure S8). It is known that until P11, the cell bodies of cone photoreceptors in the mouse are normally dispersed throughout the ONL, only to relocate subsequently to the scleral edge of the ONL around P12 [23]. It is possible that in the *rd7* mutant retina, there is a short delay in the relocation of the M-opsin–expressing cone cell bodies to the scleral edge of the ONL.

### Rod Genes Are Only Modestly and Temporarily Affected in *rd7*

In sharp contrast to changes in cone gene expression, rod-specific genes were much less severely affected in the *rd7* mutant. Microarray and in situ hybridization analysis of numerous rod genes failed to reveal marked changes in expression levels at P14 and P21 (see Figure 2, lower left photomicrographs; Table S2). In addition to the three rod genes depicted in Figure 2, in situ hybridization analysis on an additional 19 rod-specific and pan-photoreceptor genes demonstrated only a very mild diminution of expression in two of these genes, *gucy2e* and *Rgs9,* at P14, and an increase in expression in two, *Nr2e3,* and *Cnga1* (Table S2).

**Figure 6:**
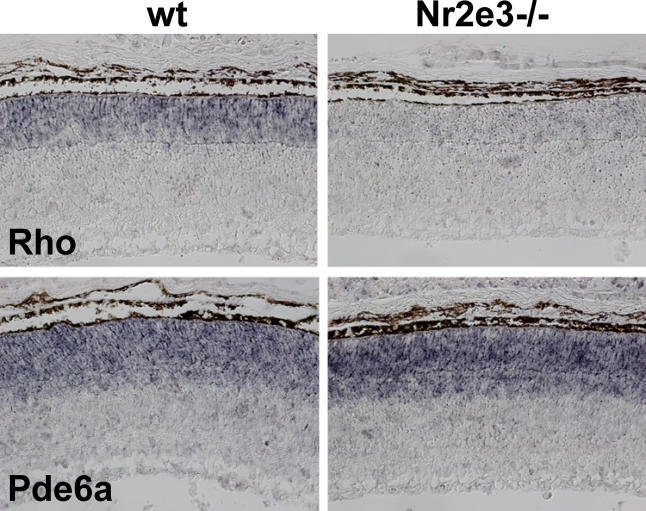
The Onset of *rhodopsin* Expression Is Delayed in the *rd7* Mutant Retina Note the nearly undetectable staining for *rhodopsin* in this P6 mutant retina (top right). The majority of rod-specific genes did not show this delay in expression onset, as indicated by the normal amount of staining for *Pde6a* in the mutant at P6 (bottom images).

Despite the minimal changes in rod gene expression at later postnatal timepoints, there was evidence of a significant delay in the onset of *rhodopsin* (*Rho)* expression in *rd7* mutants relative to wild-type. Microarray analysis at P6 demonstrated five cDNA spots that were down-regulated in three out of three experiments. Of these spots, three corresponded to *rhodopsin* (Table S3). In situ hybridization analysis of several rod-specific genes at P6 revealed that *rhodopsin* alone showed a markedly lower level of expression compared to wild type (Figure 6; unpublished data). Despite this modest delay in the onset of *rhodopsin* expression, by P14 the gene had attained nearly normal levels in the *rd7* mutant (see Figure 2, lower left photomicrographs). This latter finding suggests that all the rod- and many cone-specific genes are coexpressed in the majority of photoreceptors in the *rd7* mutant.

### Changes in Retinal Transcription Factor and Müller Glial Gene Expression in *rd7*

Analysis of several photoreceptor transcription factors in the *rd7* mutant indicated that the levels of *Crx* and *Nrl* are unaffected in the mutant at P14 (see Figure 2, lower right photomicrographs). *Nrl* is a rod-specific, basic leucine zipper transcription factor required for the activation of many rod-specific genes and the repression of most cone-specific genes in rods [24]. *Nrl* is known to be genetically upstream of *Nr2e3* and is required for its expression [24]. *Crx* is a homeobox transcription factor expressed in both rods and cones and is required for the expression of a variety of rod- and cone-specific genes [25]. In contrast to what is seen in the *Nrl* mutant, *Nr2e3* expression is unchanged in *Crx* mutant homozygotes (unpublished data).

Strikingly, *Nr2e3* itself was markedly up-regulated in the *rd7* mutant both by microarray and in situ hybridization (see Figure 2, lower right photomicrographs; Table S2). The *rd7* mutant carries a deletion in *Nr2e3* that removes portions of both the DNA-binding and ligand-binding domains [15]. Although this deletion most likely creates a null allele, a residual transcript is clearly present and up-regulated in the *rd7* mutant. This finding suggests that *Nr2e3* is required for repression of its own transcription.

One gene, RIKEN cDNA 4933409K07 (Figure 1, gene G47), was found to be up-regulated on microarray at all four timepoints examined. This gene showed a unique pattern of expression in the adult *rd7* mutant retina. Whereas there was only a barely detectable hint of expression in the inner nuclear layer (INL) in the wild-type retina, this gene showed strong expression in the middle and vitreal thirds of the INL as well as patchy expression in the ganglion cell layer (GCL) and at the scleral edge of the ONL in *rd7* (see Table S1). This in situ hybridization pattern is consistent with staining in Müller glia, the principal glial cell type of the mouse retina. One possible interpretation of this unusual expression pattern is that it represents an early reaction of Müller glia to injury in this mutant.

### The Majority of the Photoreceptors in the *rd7* Retina Represent a Morphologically Hybrid Cell Type

In order to assess the morphologic effects of the above gene expression changes, the ultrastructure of the photoreceptor cell bodies in the *rd7* mutant was examined. The cell bodies were chosen for evaluation rather than the outer segments, since in the mouse, the ultrastructural differences between rod and cone somata are much greater than are the differences between the outer segments [26]. In the wild-type mouse, cone cell bodies are aligned along the scleral border of the ONL, and they are larger than those of rods. They have a smaller, more irregular mass of nuclear heterochromatin that is often broken up into multiple discrete clumps connected by thin threads. They also have more abundant electron-lucent euchromatin than rods. Lastly, they frequently have a patch of organelle-rich cytoplasm next to their nuclei, usually containing large mitochondria [26].

**Figure 7:**
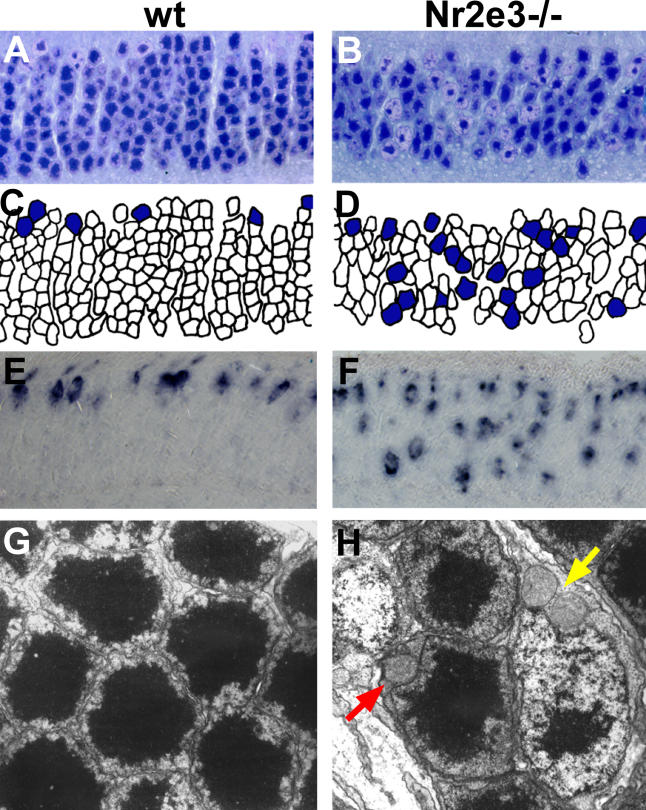
The *rd7* Mutant Retina Contains a Morphologically Hybrid Photoreceptor Cell Type in Addition to Supernumerary S-Opsin–Positive Cones (A and B) Toluidine blue-stained semi-thin sections of the outer nuclear layer (scleral edge oriented up). (C and D) Hand-drawn diagrams of the cells in (A) and (B), respectively. Cells with the nuclear features of cones are highlighted in blue. Note that the number of such cells is greater in the mutant, and their cell bodies are scattered throughout the outer nuclear layer. In addition, the overall columnar architecture of the outer nuclear layer seen in the wild type is disrupted in this portion of the mutant retina. Other portions of the mutant retina with fewer supernumerary cone cells, however, retain the normal columnar appearance (unpublished data). (E and F) Images of the outer nuclear layer (scleral edge up) stained by in situ hybridization for S-opsin. Note the typical pattern of staining at the scleral edge of the outer nuclear layer in the wild type. The *rd7* mutant retina shows supernumerary S-opsin–positive cells scattered throughout the outer nuclear layer in a distribution very similar to the supernumerary cone cells seen in (B). Since images (E) and (F) derive from different retinas than those depicted in (A) and (B), the location of the individual cells do not correspond. (G and H) Electron micrographs of the outer nuclear layer (10,000× magnification). Note the uniform distribution of rod cell bodies in the wild type (G). The cell bodies are nearly round and consist almost exclusively of a nucleus with a single, dense mass of heterochromatin. In the *rd7* mutant (H), two types of cell are shown. The ovoid one with a lesser quantity of heterochromatin, paler euchromatin, and two juxtanuclear mitochondria (yellow arrow) represents a typical cone cell body. The adjacent cell with a more “rod-like” mass of heterochromatin and a single juxtanuclear mitochondrion (red arrow) represents one of the hybrid photoreceptors discussed more fully in the main text.

Analysis of toluidine blue-stained semi-thin sections revealed that such cone-like cells were present in greater abundance in the *rd7* retina than in wild-type, and that their somata were scattered throughout the ONL (Figure 7). A comparison between the distribution of these cells and those expressing S-cone opsin strongly suggests that they represent the same cell population (compare Figure 7D and 7F). Analysis of the nuclear morphology of dissociated retinal cells stained for S-cone opsin by dissociated cell in situ hybridization confirmed that this is the case (unpublished data). These findings, along with the absence of rhodopsin staining in these cells (see Figure 3D–3F), suggest that these “cone-like” cells in the *rd7* mutant retina may represent supernumerary normal cones with an abnormal localization of their cell bodies.

In contrast to the cone cell body, the wild-type rod soma is small and nearly round. It has a single, large clump of dense heterochromatin, a thin rim of moderately electron-dense euchromatin, and very scant juxtanuclear cytoplasm without organelles [26,27]. The second cell population in the ONL of the *rd7* retina has some of the nuclear features of normal rods, such as a single, dense mass of heterochromatin and moderately electron-dense euchromatin (Figure 7H); yet these cells also show features of cones. First, the euchromatin is, on average, more abundant in these cells than in wild-type rods (compare Figure 7G and 7H). In addition, comparison of the diagrammatic representation of the wild-type and *rd7* ONLs suggests that the average area of the S-opsin–negative cells in *rd7* is greater than in the wild-type (Figure 7C and 7D). In order to confirm this impression, we quantitated the area of 50 wild-type and 50 mutant rod-like cell bodies (see Materials and Methods for details). This experiment confirmed that the average area of the rod-like somata in *rd7* is approximately 30% larger than that of wild-type rod somata (mean area in *rd7* was 9.75 ± 1.36 (standard deviation) μm^2^, compared to wild-type rods, with 7.53 ± 0.72 μm^2^ ; *n* = 50; *p* = 7.6 × 10^–16^, Student's *t*-test). It is also notable that the standard deviation of the somal area is nearly twice as great in *rd7* than in wild-type, confirming the subjective impression of greater variability in somal size and shape in the mutant compared to the wild-type (compare Figure 7C and 7D).

Lastly, 38% (19/50) of the *rd7* photoreceptors selected for somal area quantitation had prominent juxtanuclear mitochondria (red arrow in Figure 7H; unpublished data). Such juxtanuclear organelles are only very rarely seen in normal rods (1.5%; six out of 399 cells counted), but are common in cones (yellow arrow in Figure 7H). In conclusion, it is clear that this second cell population in the *rd7* retina has morphological features of both normal rods and cones consistent with the coexpression of many rod- and cone-specific genes in these cells.

## Discussion

In this paper we have determined that the primary role of the rod-specific transcription factor, Nr2e3, is to maintain cone genes transcriptionally silent within rods. We have identified two patterns of cone gene derepression in the *rd7* mutant retina, in agreement with a previous report by Chen et al. [18]. The first pattern of derepression identified (type I) consists of ectopic expression of cone genes in the vast majority of cells in the ONL. These cells were also shown to coexpress all rod genes tested. Consistent with the hybrid pattern of gene expression in these cells, electron microscopic analysis revealed them to be morphologically intermediate between normal rods and cones.

Although genes showing type I derepression demonstrated staining in the majority of cells in the ONL, two lines of evidence suggest that these genes are not completely derepressed in these cells when compared to their expression in S-opsin–expressing cones. First, close evaluation of the staining pattern of a number of type I genes in the *rd7* mutant retina (e.g., see Table S1, genes G9, G19, and G24), reveals that, in addition to the background staining throughout the ONL, there is a more darkly staining subpopulation of cells scattered throughout this layer in a distribution corresponding to that of the supernumerary S-cone opsin-expressing cells. This pattern of staining suggests that these genes are more highly expressed in S-opsin expressing cells than in the hybrid cells of the *rd7* retina.

The second line of evidence derives from a comparison of the expression pattern of many type I genes in *rd7* and *Nrl* ^−/−^ mutant backgrounds. As mentioned above, *Nrl* is a retinal transcription factor that, when mutated, results in en masse conversion of rods into S-opsin–expressing cones [24]. It can be inferred from this fact that *Nrl* is absolutely required for the normal silencing of cone-specific genes in rods. In the *Nrl* homozygous mutant, there is a stronger and more uniform derepression of many cone-specific genes throughout the ONL than is seen in the *rd7* retina (unpublished data). This finding further suggests that, in addition to its repression of cone gene expression via induction of *Nr2e3* expression, *Nrl* exerts an additional level of negative control over cone genes either directly or via a second, as yet uncharacterized, repressor.

The second pattern of derepression seen in the *rd7* retina (type II), is represented by a scattered population of cells throughout the ONL that shows derepression of several cone-specific genes, including S-cone opsin. By ultrastructural criteria, these cells appear to be normal cones, albeit with displaced cell bodies. Quantitation of these supernumerary S-cone opsin-positive cells indicates that they are approximately 2-fold more abundant than in normal retina, consistent with previous antibody studies [17].

Two recent studies have presented data that are consistent with many of the findings in our study [18,19]. Both studies showed that cone genes in addition to S-cone opsin are derepressed in the mouse *rd7* mutant. In addition, Peng et al. [19] found by RT-PCR that the levels of several rod-specific genes, including *rhodopsin,* were modestly reduced in *rd7* at P28. Our in situ hybridization data suggest that rhodopsin expression is markedly reduced at P6, but that it attains levels indistinguishable from wild-type by P14. Since the change in rhodopsin levels identified by Peng et al. were relatively small (an approximately 15% reduction), it is not surprising that such a difference was not detected by in situ hybridization. The overall finding of modest reductions in rod-specific gene expression is entirely in keeping with the results of the present study.

In addition to demonstrating derepression of a range of known cone-specific genes in *rd7* mutants, Chen et al. [18] showed up-regulation by Northern blot of two additional genes in the *rd7* mutant, *Elovl2* and *Fabp7*. These two genes were also found to be up-regulated in *rd7* in the present study (see Figure 1; Table S1). Although we found *Elovl2* to have a cone-enriched pattern of expression (see Figure 1), in situ hybridization analysis of *Fabp7* failed to show a signal in wild-type or mutant retina (unpublished data). Nevertheless, previous studies have suggested that *Fabp7* is expressed in radial glia and immature astrocytes in the brain [28–30]. Given the expression pattern elsewhere in the nervous system, it is possible that *Fabp7* is up-regulated in Müller glia in the *rd7* retina in response to injury in a manner akin to the novel Müller glial gene identified in this study, RIKEN cDNA 4933409K07 (Figure 1, gene G47). Indirect support for this idea is provided by the observation that *Fabp7* is up-regulated by microarray analysis in *Nrl* and *Crx* mutant retinas as well (unpublished data), suggesting that this change may represent a generalized response to injury in the retina rather than derepression of a cone-enriched gene.

The study by Chen et al. [18] made two further observations worthy of note. First, they identified a zebrafish homolog of *Nr2e3* and showed it to be expressed in photoreceptors. Interestingly, they showed that this homolog appears to have a pan-photoreceptor pattern of expression early in development that later resolves into a rod-specific pattern of expression. This early expression in cones may represent a mechanism whereby the expression of cone-specific gene products is temporarily repressed. It will be important to determine the extent to which the function of *Nr2e3* has been conserved in the retina of such a distantly related organism. Secondly, Chen et al. [18] used an in vitro oligonucleotide selection protocol to determine the preferred binding site for *Nr2e3*. This information will be very useful for future bioinformatic analyses of *Nr2e3* target genes.

**Figure 8:**
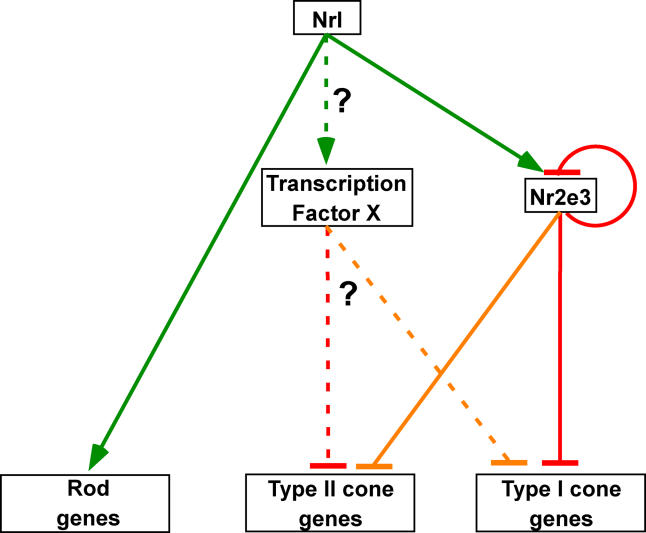
A Partial View of the Rod Photoreceptor Transcriptional Regulatory Network Note that green lines indicate activation, and yellow and red lines indicate weak and strong repression, respectively. The dotted lines associated with a question mark indicate that it is not known whether Nrl directly represses the target genes in question or whether its repression is mediated by a downstream transcription factor (“X”). Note that *Nr2e3* appears to negatively regulate its own transcription. The regulatory linkages depicted in this diagram are not necessarily direct. The weak activation of some rod-specific genes by *Nr2e3* is omitted from this diagram for clarity. Also not shown is the role of other photoreceptor transcription factors, such as *Crx*.

The gene expression changes identified in the *rd7* mutant retina in the present study suggest the scheme of gene regulation in mouse rods depicted in Figure 8. As this diagram implies, there appear to be at least two different repressors of cone genes within rods, Nr2e3 and either Nrl itself or an additional unknown transcription factor downstream of Nrl, here termed “transcription factor X.” In fact, it appears that the differences between type I and type II cone genes may simply depend on which repressor—Nr2e3 or transcription factor X—is primarily responsible for the regulation of the gene in question. In the case of type I genes, Nr2e3 is the primary repressor and transcription factor X is of secondary importance. In the case of type II genes, transcription factor X exerts the major repressive effect on transcription, and Nr2e3 plays a lesser, but still important role.

In contrast to the marked derepression of the vast majority of cone-specific genes in the *rd7* mutant, two genes stand out as being unaffected by the mutation: the gene encoding M-opsin and *Thrb2*. As *Thrb2* is known to be required for the expression of M-opsin [22], the absence of supernumerary M-opsin–positive cells may simply be a consequence of the fact that *Thrb2* expression is unchanged in the *rd7* mutant. Further support for this idea has been provided by recent work in our lab showing widespread derepression of cone genes in the *Notch1* ^−/−^ retina (unpublished data). In contrast to the *rd7* mutant, *Notch1* ^−/−^ retinas show marked derepression of *Thrb2* and a corresponding derepression of the gene that encodes M-opsin. An additional observation suggesting that M-opsin and S-opsin are controlled by different mechanisms comes from in vitro experiments [31,32]. While explanted P3 retinas express S-opsin and M-opsin with normal kinetics, explanted P0 retinas express only S-opsin [32]. The factor(s) controlling these differences are unknown, but may be intrinsic, as cocultures of older and younger retinas, conditioned media from older retinas, and addition of a variety of small molecules were unable to promote the expression of M-opsin in the P0-initiated cultures [32].

In contrast to our findings, Peng et al. [19] reported that M-opsin expression is mildly increased in the *rd7* mutant retina. It is possible that a subtle increase in M-opsin transcript levels does occur in the *rd7* retina, and that this difference could not be detected by in situ hybridization. Since virtually all M-opsin–expressing cells are localized at the outer edge of the ONL by P28 in the *rd7* mutant (Figure S8), any increase in M-opsin transcript in the mutant must have occurred in cells in that location.

A variety of novel cone-specific or cone-enriched genes were characterized in this study. One of these genes, *Pygm,* is involved in glycogen/glucose metabolism, and a second, *Glo1,* is required for detoxification of methylglyoxal, a byproduct of glycolysis [33]. A third gene involved in glucose metabolism, *hexokinase 2* *(Hk2),* is also derepressed in the *rd7* mutant and shows a pattern of expression in the wild-type retina, suggesting greater expression in cones than in rods (see Figure 1; Table S1). A fourth gene involved in glucose metabolism, *glucokinase regulatory protein (Gckr),* was found to be increased in three out of three microarrays at P21 but was not tested by in situ hybridization (Table S4). The increased expression of *Gckr* in *rd7* mutant retina suggests that it too may be a cone-enriched gene. A previous study found that two of these genes, *Pygm* and *Hk2,* have markedly elevated tag levels in an ONL-specific serial analysis of gene expression library consistent with their being highly enriched in wild-type photoreceptors [34]. Furthermore, prior studies have suggested differences in glycogen and glucose metabolism between primate rods and cones [35]. Our findings lend further support to this concept. Interestingly, *Pygm* has been implicated in human disease. Mutations in this gene underlie McArdle's disease (glycogen storage disease type V), the symptoms of which include exercise intolerance, muscle cramps, and myoglobinuria [36]. To our knowledge, no abnormalities of retinal function have been reported.

One of the most curious findings in the *rd7* mutant retina was the occurrence of two different types of changes: an increase in the number of S-opsin–expressing cones and a transformation of rods into hybrid photoreceptors. It is known that *Nr2e3* is expressed only in rods, and the transcript is first detectable in postmitotic cells (J. Trimarchi and CLC, unpublished data). Assuming that Nr2e3 acts cell autonomously, we can conclude that the supernumerary S-cone–positive cells and the hybrid photoreceptors identified in the *rd7* retina were redirected to these fates from postmitotic cells that were destined to become rods. This conclusion raises this question: Why does loss of a single transcription factor within rod precursors lead to two alternative fates—a hybrid cell type on the one hand and apparently normal S-cones on the other? There are at least two possible explanations for these differences.

First, it is possible that there are two distinct types of rod precursor; loss of *Nr2e3* in one leads to S-cone fate and in the other results in a hybrid cell type. In fact, there is experimental evidence from the rat to support the conclusion that early-born and late-born rods are intrinsically different [37]. One test of the hypothesis that there are two temporally distinct rod precursor populations would be to carry out birthdating experiments to determine whether the supernumerary S-opsin–positive cells in the *rd7* retina derived exclusively from an early- or late-born population. Of course, if this were not the case, this experiment could not rule out the possibility that molecularly distinct populations of rod precursors are present simultaneously in the developing retina.

An alternative explanation would be that there is only a single, homogeneous population of postmitotic rod precursors in the mouse, and a stochastic event triggers assumption of the S-cone fate in a small subpopulation of these cells in the *rd7* mutant. Recent studies in a variety of experiment systems suggest that such a stochastic, all-or-none mechanism of gene activation is commonplace [38–44]. In this scenario, the absence of Nr2e3 would alter the probability that an unknown master control gene is expressed in rod precursors. Once this event takes place, it would initiate an irreversible program of differentiation toward S-cone fate, albeit at a relatively low frequency. In this way, a subset of cells from an initially homogeneous population would select the S-cone fate in an entirely probabilistic manner.

Human patients with ESCS display three types of abnormality attributable to the retina: (1) an atypical ERG waveform that is preferentially sensitive to short-wavelength light, (2) slowly progressive retinal degeneration, and (3) abnormal retinal lamination with rosette formation [1,12,13]. The *rd7* mutant mice also demonstrate the latter two defects, but have a normal ERG [15,45]. These similarities and differences between the two species help to explain the possible mechanistic basis of the ESCS.

The fact that the *rd7* mouse has a normal ERG strongly suggests that the aberrant ERG in ESCS is not attributable to the activity of a hybrid photoreceptor identical to that found in this study. Namely, the signal is unlikely to derive from a population of cells coexpressing both rod and cone genes but whose photopigment is rhodopsin and not S-cone opsin. This conclusion is consistent with the evidence from human ESCS patients indicating a markedly reduced rod system and a lack of measurable rhodopsin by reflection densitometry [1,2,10,11]. It is also unlikely that we would fail to detect an ESCS-like ERG signal in mice if it were present, as such a signal has been demonstrated in the *Nrl* mutant mouse, which has a near total transformation of all its rods into blue cones [24].

These findings, however, do not rule out the possibility that the abnormal human ERG derives from a hybrid photoreceptor cell type that also expresses S-opsin. It is possible that there are gene regulatory differences between mice and humans such that in human *NR2E3* mutants, S-opsin shows a type I pattern of derepression rather than a type II as in seen in the *rd7* mouse, and is therefore expressed in all of the hybrid photoreceptor cells. Alternatively, the ratio of supernumerary S-cones to hybrid photoreceptors produced in the retina of ESCS patients might be such that a higher percentage of the presumptive rods in ESCS patients become S-cones rather than hybrid photoreceptors. As discussed above, this ratio could depend either on the relative percentages of two distinct rod precursor populations or on stochastic effects on regulatory gene expression.

In contrast to the ERG differences between mouse *rd7* and human *NR2E3* mutants, both species demonstrate slow retinal degeneration. It is possible that this degeneration is attributable to the abnormal function of the hybrid photoreceptor cell type characterized in the present study. The coexpression of both rod and cone genes in the same cell could predispose the cell to apoptosis.

The final common feature between mouse *rd7* and human *NR2E3* mutants is the presence of an abnormally laminated retina with waviness and rosette formation in the ONL [12–15]. The cause of this abnormality is not known, but it is possibly related to defects in photoreceptor cell polarity in the *rd7* mutant. Rosette formation and abnormally wavy epithelia are common sequelae of defects in pathways controlling cell polarity [46,47]. In particular, loss-of-function mutations in the polarity gene *crumbs* *(CRB1)* have been shown to cause morphological abnormalities of the ONL in both humans and mice, including rosette formation in mice very similar to that seen in the *rd7* mutant [48,49]. Interestingly, Sharon et al. [5] have recently pointed out additional features shared by patients with *CRB1* mutations and mutations in *NR2E3,* including hyperopic refractive errors and a distinctive pattern of clumped pigmentation in the retina.

In the present study we found the mouse *crumbs* ortholog to be up-regulated in the *rd7* mutant retina by microarray, consistent with its higher expression level in cones than in rods [50]. Although we were unable to confirm this finding by in situ hybridization due to the weakness of the signal, it is possible that the up-regulation of crumbs in the retina is the cause of the lamination defects seen in the *rd7* mutant. Overexpression of wild-type *crumbs* in *Drosophila* has been shown to cause polarity defects leading to waviness of epithelia and even to misalignment of nuclei within photoreceptors analogous to what is seen in the *rd7* retina [47,51]. Future experiments will address this question by overexpressing full-length *Crb1* in a wild-type background.

One further point worthy of note is the striking similarity between the hybrid photoreceptor identified in this study and a naturally occurring photoreceptor found in ground squirrels. The “rods” of this species have electrophysiologic, molecular, and ultrastructural features of both rods and cones [52–58]. Although these unusual findings have been difficult to interpret under the usual assumptions of “duplicity theory” [56], we would like to suggest that ground squirrels may have experienced a naturally occurring down-regulation or loss of *Nr2e3* expression in their “rods” that transformed them into a hybrid photoreceptor cell type. The adaptive significance of such a change, if any, is unknown, and it may simply be due to relaxation of selective pressure for night vision in this strictly diurnal species.

## Materials and Methods

### Mutant mice.

*Nr2e3^rd7^* mutant mice were obtained from Jackson Laboratories (Bar Harbor, Maine, United States; stock #004643) and maintained on a C57BL/6 background. All control mice were C57BL/6.

### Microarray analysis.

Total retinal RNA samples were isolated from P0, P6, P14, and P21 *Nr2e3* mutant mice using the Trizol reagent (Gibco, San Diego, California. United States). Total retinal RNA samples from age-matched wild-type C57BL/6 mice were used as controls. Individual total RNA samples were derived from four retinas (pooled from two animals). All microarray experiments were performed in triplicate, in each case with separate RNA preparations. Microarray experiments with cDNAs were performed with the P0, P6, and P14 derived samples. Probes were labeled with either Cy3 or Cy5 using the Array 900 kit from Genisphere (Hatfield, Pennsylvania, United States) starting with 5 μg of total RNA according to the manufacturer's instructions. Wild-type control probes were compared to mutant on the same microarray. In two of the three replicates, the mutant probe was labeled with Cy3 and the wild type with Cy5, and in the third replicate the dyes were swapped. Labeled probe was hybridized to microarray slides spotted with approximately 11,500 cDNA clones from the brain molecular anatomy project library (kind gift of B. Soares, University of Iowa; see http://trans.nih.gov/bmap/index.htm for details) and 500 cDNA clones from our lab collection. Slides were printed and hybridized as described [59,60]. After hybridization and washing of slides according to the manufacturer's instructions (Genisphere), the slides were scanned on an Axon Instruments (Union City, California, United States) GenePix 4000 scanner and images were analyzed using the accompanying GenePix Pro software package. The complete raw cDNA microarray data set are available in Tables S6–S14.

Two types of normalization were performed on the raw intensity scores derived from the GenePix Pro analysis. First, for a given experiment, the average intensity of all the spots in the weaker of the two channels (Cy3 or Cy5) was normalized to those in the stronger channel. Second, in a given set of experiments done in triplicate at a particular time point, the two experiments with the weaker average signal intensity over all spots were normalized to those in the third microarray with the strongest average signal intensity. All spots with signal levels equal to or below background were removed from the analysis. The resulting files contained on average about 6,000 spots. These files were then sorted according to Cy3/Cy5 signal intensity, and those spots with the 10% highest and 10% lowest intensity ratios (approximately 600 spots/experiment) were compared across the three experiments at a given time point using custom Perl scripts (available upon request from JCC). All spots which were present in the top 10% most up- or down-regulated genes in two out of three or three out of three experiments were recorded (the latter are listed in Table S3).

Microarray analysis of the P21 retinas was performed on Affymetrix mouse genome 430 2.0 GeneChip array (Affymetrix, Santa Clara, California, United States). A total of six microarray hybridizations were performed: three with probes derived from mutant RNA and three from wild-type. Probes were synthesized starting with 10 μg of total RNA for each sample according to manufacturer's instructions (Affymetrix). Hybridization, washing, and scanning of the microarrays were all performed at the Bauer Center for Genomics Research at Harvard University according to manufacturer's instructions (Affymetrix). Initial data analysis was carried out using the GeneChip Operating System (GCOS) software from Affymetrix. Pairwise comparisons were made between individual mutant microarray results and controls. All genes were removed from the analysis for which “absent” calls were made by the software for both the wild-type and mutant samples being compared. The remaining gene lists contained approximately 26,000 transcripts. These lists were then sorted according to the mutant-to-wild-type “signal log ratio” in order to identify the most markedly up- and down-regulated genes. The top 500 most up- and down-regulated transcripts (approximately 2% of all genes in each case) from each of the three pairwise comparisons between mutant and wild-type were compared using custom Perl scripts (available upon request from JCC) to identify those genes that were present in two or three out of three lists. Those genes that were up- or down-regulated in three out of three experiments were recorded (Tables S4 and S5). The complete pairwise Affymetrix microarray datasets are available in Tables S15–S17.

### In Situ hybridization.

Section in situ hybridization was performed as previously described [61] using 20-μm cryosections from OCT-embedded tissue or 4-μm paraffin sections. All in situ hybridizations were performed with the mutant and wild-type control sections on the same glass slide. Riboprobes labeled with digoxygenin-tagged UTP (Roche, Basel, Switzerland) were detected with NBT/BCIP (Sigma, St. Louis, Missouri, United States). The sources of the individual riboprobes used in this study are described in Tables S1 and S2. Dissociated cell in situ hybridization was performed as described previously [62] using the same S-opsin digoxygenin-labeled probe used for section in situ hybridization. All images were captured on a compound microscope (Eclipse e1000; Nikon, Tokyo, Japan) using a CCD camera (DXM1200F, Nikon). S-opsin positive cells were quantitated on dissociated cell in situ hybridization as previously described [62]. Twenty fields were quantitated in this manner at 200× magnification for both *rd7* and wild-type retinas.

### Immunohistochemistry.

For antibody staining, cryosections were prepared and stained as described previously [63]. Primary antibodies used were a polyclonal anti-blue opsin raised in rabbit (1:300; Chemicon International, Temecula, California, United States; AB5407) and a mouse monoclonal anti-rhodopsin (1:200; rho4D2). Secondary antibodies used were Cy2- or Cy3-conjugated goat anti-rabbit and anti-mouse (1:500; Jackson Immunologicals, West Grove, Pennsylvania, United States). Following antibody staining, 4′-DAPI was applied to stain nuclei (Sigma), and the sections were coverslipped and mounted in Gel/Mount (Biomeda, Foster City, California, United States).

### Electron microscopy.

This protocol was adapted from one used by Raviola [64] with some modifications derived from Carter-Dawson and Lavail [26]. Four adult wild-type and four mutant animals were deeply anesthetized by intraperitoneal injection of Avertin and the eyes were then removed. The cornea was gently punctured with sharp forceps and excised with iridectomy scissors. The eye was then transferred to a solution of 2% paraformaldehyde/2% glutaraldehyde in cacodylate buffer (0.1 M cacodylic acid; 0.1% calcium chloride). The lens was gently removed and the eyecup allowed to fix for 2 h at room temperature. The fixed eye was then placed on dental wax and sectioned in the midline with a fresh razor blade (half of the retinas were sectioned along the D-V axis and half along the nasal-temporal axis). The retinas (and attached retinal pigment epithelium) were carefully dissected away from the sclera, which was discarded.

The retinas were then rinsed four times for 15 min each with Sorenson's buffer (pH 7.4). They were then stained for 2 h at 4 °C with 1% osmium tetroxide in 1.5% potassium ferrocyanide. Next, the retinas were rinsed four times for 15 min in maleate buffer (pH 5.1) and then stained for 2 h at room temperature with 1% uranyl acetate in maleate buffer (pH 6.2). They were then washed four times for 15 min with maleate buffer (pH 5.1); once for 10 min with 70% ethanol; once for 10 min with 95% ethanol; and four times for 30 min with 100% ethanol. Next, the retinas were washed three times over one hour with propylene oxide and then embedded in TAAB 812 Resin (Marivac, Quebec, Canada) for 1–2 d in a 60 °C oven. Semi-thin sections were cut at a thickness of 0.5 μm and stained with 1% toluidine blue in 1% sodium borate buffer. Images of semi-thin sections from the mutant retina were taken within the ventral two-thirds of the retina where the majority of the supernumerary S-opsin–expressing cells reside. Wild-type images were taken in comparable regions. Sections for electron microscopy were cut at a thickness of 95 nm, placed on grids, and poststained with 2% uranyl acetate followed by 0.2% lead citrate. All sections were cut in a plane perpendicular to the plane of the photoreceptor layer. They were then visualized on a Jeol 1200EX electron microscope (Jeol, Tokyo, Japan). The electron microscopic images in Figure 7 derive from the ventral two-thirds of the wild-type and mutant retinas.

The area of the cell bodies of the rods and “rod-like” cells in the wild-type and mutant ONLs, respectively, were quantitated in the following manner. First, ten fields within the ONL were chosen at random at 1,000× magnification and then photographed at 4,000× magnification for each of the two genotypes. Such images typically contained 35–45 cell bodies. In order to quantitate only the area of those cell bodies that were cut as near to the midline of the cell as possible (i.e., in order to obtain the maximal cross-sectional area) the five cells with the largest apparent area in each photograph were chosen by eye and the outline of the cell membranes were traced onto white paper. These tracings were scanned along with the size bar from the electron micrographs, and the areas of the resulting digital images were quantitated using Scion Image software (NIH Image, http://rsb.info.nih.gov/nih-image). A total of 50 cells of each genotype were quantitated in this manner. In addition, in order to evaluate the percentage of rods in the wild-type retina that had any juxtanuclear mitochondria, all rods within all ten images were counted as were the number of cells showing juxtanuclear organelles. A total of six out of 399 wild-type rods (1.5%) possessed a juxtanuclear organelle. This analysis permitted us to evaluate the wild-type rods for juxtanuclear mitochondria at multiple planes of section; however, serial sections of individual cell bodies were not performed.

## Supporting Information

Figures S1–S7 show the in situ hybridization images of all genes discussed in the paper (see Tables S1 and S2). All paired images (which show the wild-type control on the left and the *rd7* mutant retina on the right) are labeled in the lower left-hand corner with the gene symbol followed by the age of the retinas in question (P6, P14, P28, or adult). Unpaired images represent prenatal time points and are labeled with the gene symbol of the gene in question (“wt” indicates that the retina is from a wild-type animal) and a designation of the embryonic day from which the retina derives (e.g., e17.5 = embryonic day 17.5).

Figure S1 In Situ Hybridization Images for G1–G13 in Table S1
(3.1 MB JPG)

Figure S2 In Situ Hybridization Images for G14–G25 in Table S1
(2.4 MB JPG)

Figure S3 In Situ Hybridization Images for G26–G35 in Table S1
(2.1 MB JPG)

Figure S4 In Situ Hybridization Images for G36–G46 in Table S1
(2.0 MB JPG)

Figure S5 In Situ Hybridization Images for G47–G53 in Table S1
(1.7 MB JPG)

Figure S6 In Situ Hybridization Images for Genes 1–11 in Table S2
(2.9 MB JPG)

Figure S7 In Situ Hybridization Images for Genes 12–22 in Table S2
(3.2 MB JPG)

Figure S8 In Situ Hybridization Results for M-Opsin and *Thrb2*
Note that the M-opsin–positive cells are scattered throughout the ONL at P14, but appear to have migrated to the scleral edge of the ONL by P28. There is no change in the number of M-opsin– or *Thrb2*-positive cells in the *rd7* mutant.
(2.1 MB PDF)

Table S1 Cone-Specific and Cone-Enriched Genes Evaluated in the *rd7* Mutant by Microarray and In Situ Hybridization This table is a supplemental version of Figure 1. “Figure Number” indicates which figure (Figure S1–S5) contains the in situ hybridization images corresponding to the gene in question. “Lab Clone Information” indicates the region of the gene in question from which the probe used for in situ hybridization was derived. All abbreviations are as indicated in Figure 1.
(26 KB XLS)

Table S2 Rod Genes Evaluated in the *rd7* Mutant by In Situ Hybridization This table contains details about the in situ hybridization patterns of 22 genes (many of which are rod-specific) evaluated in the *rd7* mutant retina. “Figure Number” indicates which figure (Figure S6 or S7) contains the in situ hybridization images corresponding to the gene in question. “Lab Clone Information” indicates the region of the gene in question from which the probe used for in situ hybridization was derived. The color coding of the in situ hybridization results under “In Situ Pattern” is as follows: dark green, markedly down-regulated; light green, mildly down-regulated; red, markedly up-regulated; orange, mildly up-regulated.
(20 KB XLS)

Table S3 Summary of cDNA Microarray Results from P0, P6, and P14 The spots listed in this table represent those that were either up- or down-regulated in three out of three microarray experiments as described in Materials and Methods. The Cy3/Cy5 signal ratios are indicated for all three microarray experiments at each time point. Note that the Cy3/Cy5 ratios for “Microarray #3” are reversed relative to the other two, since the fluorescent tag used to label wild-type and mutant RNA was swapped as described in Materials and Methods.
(22 KB XLS)

Table S4 Summary of Genes Up-Regulated in *rd7* Mutant Retina at P21 by Affymetrix Microarray Only genes that were found to be up-regulated in three out of three microarray experiments (as described in Materials and Methods) are listed. “Nr2e3 signal” and “C57BL/6 signal” represent the average signal for that transcript in all three microarray experiments.
(92 KB XLS)

Table S5 Summary of Genes Down-Regulated in *rd7* Mutant Retina at P21 by Affymetrix Microarray Only genes that were found to be down-regulated in three out of three microarray experiments (as described in Materials and Methods) are listed. “Nr2e3 signal” and “C57BL/6 signal” represent the average signal for that transcript in all three microarray experiments.
(58 KB XLS)

Table S6 Raw cDNA Microarray Data for *rd7* versus Wild-Type Comparison at P0 (I) (5.6 MB XLS)

Table S7 Raw cDNA Microarray Data for *rd7* versus Wild-Type Comparison at P0 (II) (5.6 MB XLS)

Table S8 Raw cDNA Microarray Data for *rd7* versus Wild-Type Comparison at P0 (III) (5.6 MB XLS)

Table S9 Raw cDNA Microarray Data for *rd7* versus Wild-Type Comparison at P6 (I) (5.6 MB XLS)

Table S10 Raw cDNA Microarray Data for *rd7* versus Wild-Type Comparison at P6 (II) (5.6 MB XLS)

Table S11 Raw cDNA Microarray Data for *rd7* versus Wild-Type Comparison at P6 (III) (5.6 MB XLS)

Table S12 Raw cDNA Microarray Data for *rd7* versus Wild-Type Comparison at P14 (I) (5.5 MB XLS)

Table S13 Raw cDNA Microarray Data for *rd7* versus Wild-Type Comparison at P14 (II) (5.6 MB XLS)

Table S14 Raw cDNA Microarray Data for *rd7* versus Wild-Type Comparison at P14 (III) (5.6 MB XLS)

Table S15 Raw Affymetrix Microarray Data for *rd7* versus Wild-Type Comparison at P21 (I) (18 MB XLS)

Table S16 Raw Affymetrix Microarray Data for *rd7* versus Wild-Type Comparison at P21 (II) (18 MB XLS)

Table S17 Raw Affymetrix Microarray Data for *rd7* versus Wild-Type Comparison at P21 (III) (18 MB XLS)

## Abbreviations

DAPI: 6-diamidino-2-phenylindole
E[number]: embryonic day [number]
ERG: electroretinogram
ESCS: enhanced S-cone syndrome
GCL: ganglion cell layer
INL: inner nuclear layer
ONL: outer nuclear layer
P[number]: postnatal day [number]
