# Correction: A Hybrid Photoreceptor Expressing Both Rod and Cone Genes in a Mouse Model of Enhanced S-Cone Syndrome

**DOI:** 10.1371/journal.pgen.0040018

**Published:** 2008-01-25

**Authors:** Joseph C Corbo, Constance L Cepko

Correction for:

Corbo JC, Cepko CL (2005) A hybrid photoreceptor expressing both rod and cone genes in a mouse model of enhanced S-cone syndrome. PLoS Genet 1(2): e11. doi:10.1371/journal.pgen.0010011

Resequencing has shown that three clones used for making in situ hybridization (ISH) probes for this manuscript were initially misidentified (gene IDs from Figure 1: G17, G23, and G25). The correct identities of these clones are as follow: *Glo1*, *Hnrpa1*, and *Dkk3*, respectively. This finding led the authors to resynthesize and test all ten probes made in the batch which included these misidentified probes (gene IDs G16-G25). They found that eight of the ten resynthesized probes failed to produce a cone-specific pattern of staining. The probe synthesis protocol used in the manuscript included a PCR amplification step. The authors therefore reason that the original batch of PCR reactions was subject to trace contamination by a strong cone-specific product (possibly deriving from *Opn1sw*), which resulted in spurious cone-specific staining. In order to remove the possibility of PCR contamination, all ten probes were resynthesized from restriction enzyme-linearized templates. Again, only two probes, *Glo1* and *4921511K06Rik* (Gene IDs G17 and G24), showed cone-specific staining. Further analysis of other probes used in this study has confirmed that they were free of such contamination. These findings do not alter any of the manuscript's fundamental conclusions.

