# Supplementary material for: A Hybrid Photoreceptor Expressing Both Rod and Cone Genes in a Mouse Model of Enhanced S-Cone Syndrome
Source: PLoS Genet. 2005 Aug 5;1(2):e11. doi: 10.1371/journal.pgen.0010011 (PMC1186732; doi:10.1371/journal.pgen.0010011)
Supplement: Supplementary file 1 [file pgen.0010011.sg008.pdf]

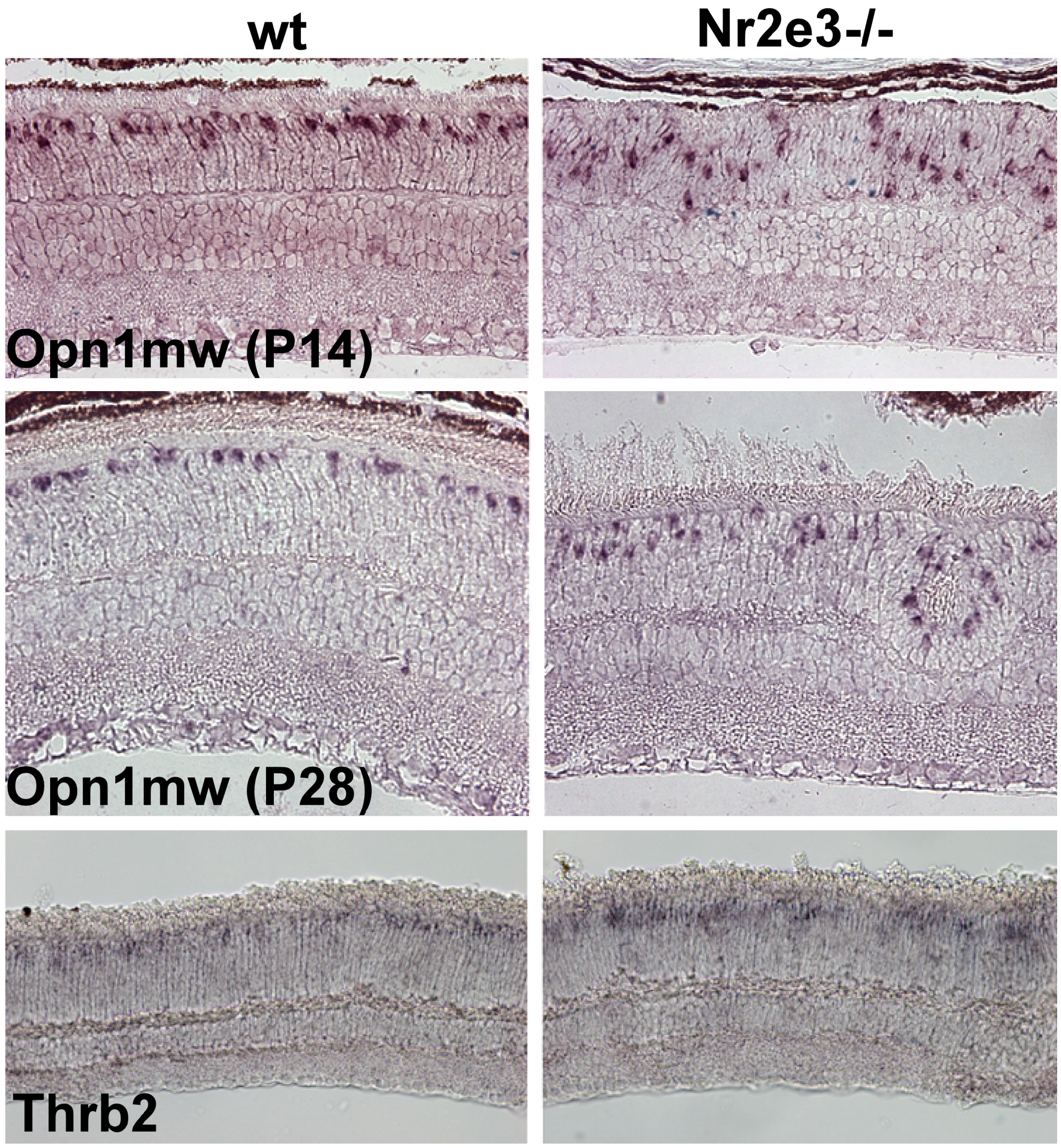
wt
Nr2e3-/-
Opn1mw (P14)
Opn1mw (P28)
Thrb2
